# Risk factors associated with major adverse cardiac and cerebrovascular events following percutaneous coronary intervention: a 10-year follow-up comparing random survival forest and Cox proportional-hazards model

**DOI:** 10.1186/s12872-020-01834-1

**Published:** 2021-01-18

**Authors:** Maryam Farhadian, Sahar Dehdar Karsidani, Azadeh Mozayanimonfared, Hossein Mahjub

**Affiliations:** 1 Research Center for Health Sciences, Department of Biostatistics, School of Public Health, Hamadan University of Medical Sciences, P.O. Box 4171-65175, Hamadan, Iran; 2 Department of Biostatistics, School of Public Health, Hamadan University of Medical Sciences, Hamadan, Iran; 3 Department of Cardiology, Medical School, Hamadan University of Medical Sciences, Hamadan, Iran

**Keywords:** Percutaneous coronary intervention, CABG, Major adverse cardiac event, Random survival forest

## Abstract

**Background:**

Due to the limited number of studies with long term follow-up of patients undergoing Percutaneous Coronary Intervention (PCI), we investigated the occurrence of Major Adverse Cardiac and Cerebrovascular Events (MACCE) during 10 years of follow-up after coronary angioplasty using Random Survival Forest (RSF) and Cox proportional hazards models.

**Methods:**

The current retrospective cohort study was performed on 220 patients (69 women and 151 men) undergoing coronary angioplasty from March 2009 to March 2012 in Farchshian Medical Center in Hamadan city, Iran. Survival time (month) as the response variable was considered from the date of angioplasty to the main endpoint or the end of the follow-up period (September 2019). To identify the factors influencing the occurrence of MACCE, the performance of Cox and RSF models were investigated in terms of C index, Integrated Brier Score (IBS) and prediction error criteria.

**Results:**

Ninety-six patients (43.7%) experienced MACCE by the end of the follow-up period, and the median survival time was estimated to be 98 months. Survival decreased from 99% during the first year to 39% at 10 years' follow-up. By applying the Cox model, the predictors were identified as follows: age (HR = 1.03, 95% CI 1.01–1.05), diabetes (HR = 2.17, 95% CI 1.29–3.66), smoking (HR = 2.41, 95% CI 1.46–3.98), and stent length (HR = 1.74, 95% CI 1.11–2.75). The predictive performance was slightly better by the RSF model (IBS of 0.124 vs. 0.135, C index of 0.648 vs. 0.626 and out-of-bag error rate of 0.352 vs. 0.374 for RSF). In addition to age, diabetes, smoking, and stent length, RSF also included coronary artery disease (acute or chronic) and hyperlipidemia as the most important variables.

**Conclusion:**

Machine-learning prediction models such as RSF showed better performance than the Cox proportional hazards model for the prediction of MACCE during long-term follow-up after PCI.

## Background

Cardiovascular disease represents a considerable health problem and is a major cause of death worldwide [1]. The condition is commonly treated with Percutaneous Coronary Intervention (PCI), which is a low-cost procedure as compared to coronary artery bypass graft surgery (CABG), requiring shorter hospitalization and recovery times. Deployment of drug eluting stents (DES) have largely replaced the use of bare metal stents (BMS), improving long-term prognosis, mainly by reducing the rate of restenosis [2]. Until now, identifying potential risk factors for subsequent major adverse cardiovascular events may offer additional advantages with respect to outcome [2–5]. However, this requires suitable models for determining the risk factors.

The Cox model is a general quasi-parametric choice for analyzing censored data. This model relates the log of the hazard ratio to a linear function of the predictors. There have been several limitations for the Cox model such as requiring medical knowledge to model covariate interaction in terms of complex nonlinear forms, as well as the proportional hazard assumption [6, 7]. Failure to establish and ignore these assumptions can affect the validity of the results.

Random Survival Forest (RSF), as an ensemble learning method, has been developed to overcome the problems mentioned in the Cox model and other classical models for the analysis of survival data. The most important feature of RSF is the proper performance of this model for measuring the importance of variables [8]. This model is also suitable for medical research in the field of high dimensional data [9–11]. Various studies have evaluated the performance of the RSF model in comparison with the Cox model [12].

Several studies have been performed on the risk factors of future adverse events following PCI with the use of BMS and DES [5, 13]. However, there are a limited number of studies describing the results of long term follow up after PCI treatment, and results from long-term follow-up may not necessarily match those of short-term follow-up. Furthermore, to the best of our knowledge, the RSF model has not previously been used to identify factors affecting the occurrence of MACCE in patients undergoing angioplasty with stent deployment.

Therefore, we have conducted a long-term study to identify factors affecting the occurrence of MACCE following coronary stenting, comparing the RSF and Cox proportional-hazards models.

## Methods

The current retrospective cohort study was performed on 220 patients (69 women and 151 men) undergoing coronary angioplasty from March 2009 to March 2012 in Farshchian Medical Center in Hamadan city, Iran. In this study, major adverse cardiovascular and cerebrovascular events known as MACCE were selected as the designated events (including death, CABG, stroke and repeat revascularization) for survival analysis.

Survival time (months), as the response variable, was considered from the date of angioplasty to the end of the follow-up period (September 2019) or the occurrence of MACCE. For the patients who had not experienced MACCE, the time from the date of angioplasty to the end of the follow-up time was considered as the censored survival time.

To identify the factors influencing the occurrence of MACCE during 10 years follow-up after coronary angioplasty, the performance of the Cox model and RSF model were investigated. Also, the event-free survival curve from MACCE was constructed with the Kaplan–Meier method.

It should be noted that the restricted mean survival time (RMST) reported for between-group summary metrics. Unlike median survival time, it is estimable even under heavy censoring.

### Cox proportional hazard model

Cox proportional-hazards model specifies the conditional hazard function based on the vector of predictor variables**.** The general form of hazard for the ith subject with the X_i_ profile at the time of t based on the Cox model was as follows:

$${\text{h}}\left( {{\text{t}},{\text{x}}} \right) = h_{0} \left( {\text{t}} \right)\exp \left(\sum \beta_{i} x_{i}\right)$$

The Cox model consists of two components: non-parametric component as unspecified increasing function, known as the baseline hazard (h0) and the parametric component, which is a linear and multiplicative function of the Xi [6].

### Random forest survival model

The RSF model, as a tree-based ensemble non-parametric algorithm can solve the limitations of the Cox model as well as identify and rank the most important variables affecting survival time. Ensemble learning is a type of supervised learning technique in which the main idea is producing several models in a training data set and then combining (average) output rules or the hypotheses obtained from them [14].

In general, the RSF algorithm includes the following steps:

1. The number of B Bootstrap samples were selected from the original data. In each bootstrap sample, about one-third of the data was out of the bag. For example, 1000 samples of Bootstrap were selected from the main data, in each Bootstrap sample, 670 samples were used for training, and the remaining out-of-bag (OBB) sample used for testing and estimation of prediction error.
2. A survival tree-based Nelson-Aalen estimator was grown for each Bootstrap sample. In each node of the tree, mtry covariates were randomly selected out of all *p *covariates for splitting. A variable was chosen to maximize the separation between two formed tree nodes. Growth stops after a certain stop condition is met (e.g., when the number of observations within a terminal node is less than a preset value or when the node becomes pure). Default values of mtry = √ p and the log-rank statistic are used as split criteria.
3. To obtain a risk prediction ensemble, information from the terminal nodes (nodes with no further split) of B survival trees were aggregated. For each tree, the cumulative hazard function (CHF) is calculated, and then the average of these CHFs reports the ensemble CHF.
4. The prediction error was calculated for the ensemble CHF using OOB data.

In this study, the implementation of the RSF model for data in each time consisted of 2000 trees based on log-rank as splitting criteria. The relative importance of each variable was also assessed using VIMP criteria. The larger the VIMP value for a variable, the more important the predictor role of that variable.

### Evaluation of survival models

Brier Score, as a measure to evaluate the performance of different survival models, is the mean square error of the prediction and indicates the predictive ability of a prediction model. Smaller values of the Brier Score indicate a more accurate prediction. The general form of the score is as follows:

$${\text{BS}}({\text{t}},\hat{S}) = {\text{E}}\left( {Y_{i} \left( {\text{t}} \right) - \hat{S}\left( {{\text{t}}|X_{i} } \right)} \right)^{2}$$

where $Y_{i} \left( {\text{t}} \right)$ is the event status for the i-th subject at time t, and $\hat{S}\left( {{\text{t}}|X_{i} } \right)$ is the survival probability for this person at time t according to the model [15].

Therefore, IBS (Integrated Brier Score) and C index criteria based on OOB data were used to compare the performance of Cox models and the random survival forest. It should be noted that for computing the evaluation criteria, all variables were included in both models.

Analyses were performed using the R3.6.3 (randomForestSRC, pec, survival) software package. The significance level was considered as 5%.

## Results

From March 2009 to March 2012, 220 patients, including 151 males (66.8%) and 69 females (31.4%) who underwent PCI with stents implantation, were retrospectively evaluated. During angioplasty, the mean age of patients was 60.11 ± 11.09, which in the males (58.74 ± 11.07) was statistically shorter than that of the females (62.75 ± 10.72) (*P* = 0.013). Table 1 presents descriptive information of the patients and the comparison of the median survival time based on the Log Rank test for each of the variables.

**Table 1:** One to ten year survival rate for the patients who underwent angioplasty from March 2009 to March 2012 in Hamadan (west of Iran)

Interval start time (months)	Proportion surviving
0	1
12	0.991
24	0.973
36	0.918
48	0.891
60	0.831
72	0.771
84	0.682
96	0.601
108	0.470
120	0.394

**Fig. 1:**
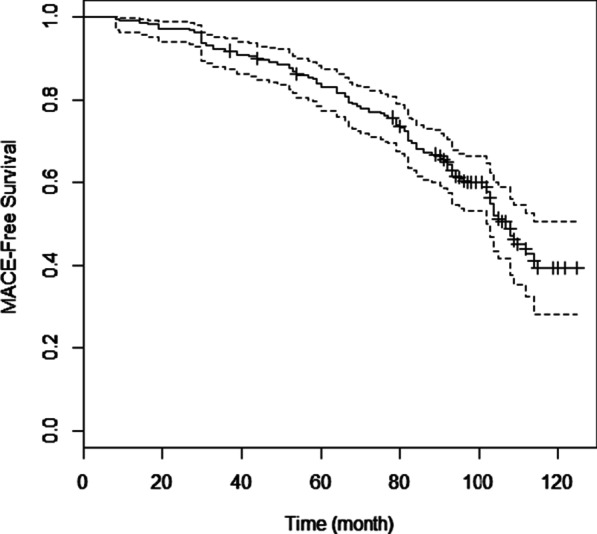
Kaplan Meier plot of MACCE free survival

During a mean follow-up period of 96.65 months, 96 (43.7%) of the 220 patients experienced MACCE. Of them, 48 patients passed away (21.8%), 16 patients (7.3%) underwent CABG, 5 patients had a non-fatal myocardial infarction (2.3%) and 27 patients (12.3%) required repeat revascularization. Most of the deaths (44 patients) are due to cardiac complications and only the cause of the death of 4 patients was reported to be cancer. The median survival time was 98 months. The 1–10 year’s survival rate is also presented in Table 2. Patient survival decreased from 99% in the first year of follow-up to 39% at the end of the follow-up. Figure 1 illustrates the survival function of patients using the Kaplan Meier method. The estimated MACCE free survival during the follow-up period was only 39%. The results showed that the mean survival time ± SE in smokers (81.25 ± 4.19 months) was shorter than that of non-smokers (102.19 ± 2.52). Also, diabetic patients had a shorter mean survival time (73.73 ± 2.85) than non-diabetic patients (101.74 ± 2.41). Patients with hypertension experienced a shorter mean survival time (90.74 ± 3.45) than patients without hypertension (100.38 ± 2.95). Patients with a stent length greater than 20 mm had shorter survival time (89.79 ± 3.69) than the patients with a stent length of shorter than 20 mm (98.36 ± 2.64).

**Table 2:** Clinical characteristics of the patients who underwent angioplasty from March 2009 to March 2012 in Hamadan (west of Iran)

Variable	N	%	Restricted mean ± SE	Median	*P* value*
All	220	100			
Age (years)	60.00 ± 11.09				
Sex					0.903
Female	69	31.4	95.01 ± 3.96	104.00	
Male	151	68.6	96.66 ± 2.78	108.00	
Smoking					< 0.001
No	63	28.6	102.19 ± 2.52	112.00	
Yes	157	71.4	81.25 ± 4.19	93.00	
Diabetes					< 0.001
No	40	18.2	101.74 ± 2.41	114.00	
Yes	180	81.8	73.73 ± 2.85	82.00	
Hypertension					0.029
No	86	39.1	100.38 ± 2.95	114.00	
Yes	134	60.9	90.74 ± 3.45	102.00	
Hyperlipidemia					0.089
No	56	25.5	97.98 ± 2.71	109.00	
Yes	164	74.5	91.33 ± 4.17	96.00	
Stent length					0.006
≤ 20 mm	141	64.1	98.36 ± 2.64	-	
> 20 mm	79	35.9	89.79 ± 3.69	102.00	
Stent diameter					0.992
3 mm	111	50.5	96.41 ± 3.15	108.00	
3.5 mm	87	39.5	96.15 ± 3.53	108.00	
4 mm	22	10	89.68 ± 6.68	103.00	
Number of vessels					0.199
1	120	54.5	98.94 ± 3.09	108.00	
2	69	31.4	93.02 ± 3.83	103.00	
3	30	13.6	86.57 ± 4.91	86.00	
Type of stent					0.752
BMS	142	64.5	94.95 ± 3.61	103.00	
DES	78	35.5	96.92 ± 2.907	109.00	
Number of stents					0.052
1	152	69.1	95.85 ± 2.68	108.00	
2	56	25.5	98.87 ± 4.52	108.00	
3	12	5.5	81.75 ± 5.80	82.00	
PCI					0.548
Ad hoc	112	50.9	97.62 ± 3.28	112.00	
Elective	108	49.1	92.34 ± 2.74	104.00	
Setting					0.26
ACS	144	65.5	95.09 ± 2.86	105.00	
CSA	76	34.5	97.11 ± 3.58	114.00	

*Estimation is limited to the longest survival time if it is censored

**Based on the Log rank Test

**Fig. 2:**
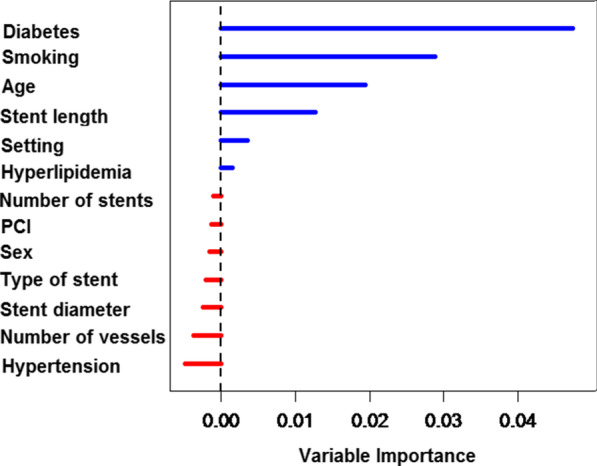
Variable importance (VIMP) of random survival forest model

The results confirmed that the proportional hazards assumption of the Cox model was generally established. The multivariable Cox model revealed that variables such as age, diabetes, smoking, and stent length had a significant effect on patient survival after angioplasty (HR = 1.03, HR = 2.17, HR = 2.41, HR = 1.74, respectively) (Table 3). Moreover, the results of comparing the Cox model and the RSF model with the log-rank splitting rule based on 2000 trees showed that the RSF model with IBS (time = 120) of 0.124 offered a better predictive performance compared to the Cox model with the value of 0.135. Also, the OOB Error Rate for the RSF was 0.352, while the OOB Error Rate for the Cox was 0.374. The C index for RSF and the Cox model was 0.648 and 0.626, respectively. According to the RSF model, the most important and influential variables affecting patient survival were diabetes, smoking, age, stent length, setting (presentation of coronary artery disease), and hyperlipidemia, respectively (as presented in Table 4 and Fig. 2).

**Table 3:** Cox regression analyses of factors associated with the occurrence of MACCE after angioplasty in a 10-year follow-up

Variable	Unadjusted hazard ratio	95% CI	*P* value	Adjusted hazard ratio	95% CI	*P* value
Diabetes			< 0.001			0.003
No	Reference			Reference		
Yes	3.01	1.95–4.64		2.17	1.29–3.66	
Smoking			0.001			< 0.001
No	Reference			Reference		
Yes	2.04	1.36–3.07		2.41	1.46–3.98	
Age (years)	1.03	1.02–1.05	< 0.001	1.03	1.01–1.05	< 0.001
Stent length			0.007			0.016
≤ 20 mm	Reference			Reference		
> 20 mm	1.73	1.15–2.58		1.74	1.107–2.75	
Setting			0.192			0.186
ACS	Reference			Reference		
CSA	1.205	0.91–1.59		0.73	0.46–1.16	
Hyperlipidemia			0.093			0.692
No	Reference			Reference		
Yes	1.44	0.94–2.21		1.106	0.66–1.83	
Number of stents			0.11			
1	Reference			Reference		
2	0.62	0.34–1.12	0.658	0.79	0.46–1.34	0.382
3	0.52	0.27–0.97	0.028	1.8	0.82–3.94	0.138
Sex			0.904			0.865
Female	Reference			Reference		
Male	0.97	0.63–1.05		1.05	0.59–1.85	
PCI			0.55			0.799
Ad hoc	Reference			Reference		
Elective	1.13	0.75–1.69		0.94	0.61–1.45	
Type of stent			0.75			0.166
BMS	Reference			Reference		
DES	0.93	0.61–1.41		1.41	0.86–2.301	
Stent diameter			0.992			0.534
3 mm	Reference			Reference		
3.5 mm	0.98	0.63–1.49	0.175	0.75	0.47–1.203	0.239
4 mm	0.97	0.47–1.97	0.187	1	0.46–2.14	0.999
Number of vessels			0.208			0.344
1	Reference			Reference		
2	1.19	0.75–1.87	0.451	1.39	0.84–2.31	0.198
3	1.64	0.94–2.87	0.078	1.45	0.77–2.74	0.245
Hypertension			0.031			0.526
No	Reference			Reference		
Yes	1.55	1.04–2.32		1.16	0.72–1.9	

*The covariates are ordered by decreasing VIMP in the RSF model

**Table 4:** VIMP of random survival forest model

	Importance	Relative importance
Diabetes	0.044	1
Smoking	0.031	0.717
Age	0.023	0.518
Stent length	0.015	0.338
Setting	0.003	0.068
Hyperlipidemia	0.001	0.032
Number of stents	− 0.001	− 0.023
Sex	− 0.001	− 0.028
PCI	− 0.0008	− 0.018
Type of stent	− 0.0007	− 0.014
Stent diameter	− 0.002	− 0.051
Number of vessels	− 0.003	− 0.073
Hypertension	− 0.003	− 0.081

In the next step of analysis confounders with significant unadjusted hazard ratios were included in the multiple cox regression. Also, for the RSF, confounders with positive VIMP were included. Then these two models were compared. The results show that for these conditions, the RSF (based on the six confounders with positive VIMP) has a better performance compared to the Cox model (based on the four confounders with significant unadjusted hazard ratios) (Table 5).

**Table 5:** Comparison of Cox regression and RSF models in different scenarios

Included variable	Model	C index	OOB error rate	IBS*
All 13 variables	Cox	0.626	0.374	0.135
	RSF	**0.648**	**0.352**	**0.124**
4 variables with significant unadjusted hazard ratios (diabetes, smoking, age, stent length)	Cox	0.670	0.328	0.126
6 variables with positive VIMP (diabetes, smoking, age, stent length, setting, hyperlipidemia	RSF	**0.697**	**0.317**	**0.112**
The same variables (diabetes, smoking, age, stent length)	Cox	0.670	0.328	0.126
	RSF	**0.684**	**0.312**	**0.113**

Bold values are the higher values of indices for comparing two models

*Integrated brier score

## Discussion

In this study, the long-term survival of cardiovascular patients after angioplasty was investigated in a 10-year follow-up. Comparing the predictive performance of the two models showed that the predictive performance of RSF was better than the Cox model.

Cox model showed that variables such as older age, diabetes, smoking, and longer stent length were the most important variables affecting patient survival. The most important factors affecting the survival of patients based on the RSF model were in order of diabetes, smoking, age, stent length, presentation of coronary artery disease, and hyperlipidemia.

To the best of our knowledge, there has been no similar study investigating the factors influencing the occurrence of MACCE after angioplasty with RSF. Until now, various studies have evaluated the short-term predictors of MACE (major adverse cardiac events) following PCI. However, few studies have focused on the long-term follow up outcomes. Most of these studies have reported short-term follow up results and compared the complications and survival of patients with DESs and BMSs.

We observed an incidence of 43.7% MACCE, whereas, Aghajan et al. [16] reported 14.4% MACE in elderly patients (with a mean age of 70.8 ± 4.7 years) during a 10 years follow-up period. During a shorter follow-up period of 2 years, Zhou et al. [17] reported 7.4% MACE, and after 3 years Meliga et al. [18] reported 26.5% MACE in patients treated at seven European and American medical centers.

Our results from both random forest and Cox's regression models showed that diabetic patients demonstrated a higher risk of MACCE (HR = 2.17). Similar results were also reported by Aghajan and coworkers (HR = 1.33), Meliga and coworkers (HR = 2.85) and Ebrahimzadeh and coworkers (HR = 2.91) [16, 18–20].

As expected, the traditional risk factors (e.g. age, diabetes and smoking) increased the risk of MACCE. One year increase in the age increased the risk of MACCE by 5%. The hazard rate of MACCE in smokers was 2.41 times that of the non-smokers. These results are consistent with the findings obtained by Farshidi et al. [21] and Tsai et al. [22], indicating a significant correlation of old age, smoking and diabetes during PCI with mortality.

The finding of this study confirmed that individuals with a stent number of 3 were 1.8 times more likely to experience MACCE than those with a stent number of 1. Also, the chance of MACCE increased with an increase in the number of involved vessels. Tsai et al. [22] found that triple vessel and stent implantation predicted the development of MACE in Chinese PCI patients.

Also, our results showed that there was no statistically significant effect of stent type on the survival of patients. The current study is observational non-randomized; therefore a comparison of two stents types will be biased according to the lesion or patient characteristic, and any interpretation of treatment result is therefore precluded (due to indication bias). However, in a randomized controlled trial study conducted by Horst et al., in patients undergoing PCI, there were no significant differences in the composite outcome of death from any cause and nonfatal spontaneous myocardial infarction between the two types of stents after a median of 5 years of follow up [23]. Flice et al., reported a statistically significant difference between the two types of stents in the occurrence of MACE during a 3-year follow-up (18% for the DES versus 28% for the BMS stent) in coronary patients with chronic obstructive pulmonary disease [24]. Cai et al., showed that there was a statistically significant difference in MACE between BMS (15.9%) and DES (8.8%) stent during 30-month follow-up [25]. Farshid et al., demonstrated that the need for re-hospitalization in patients treated with BMS was significantly higher than those treated with DES (*P* = 0.034). However, in the long-term follow-up, there was no significant difference in the mortality rate between the two types of stents [21]. Duggalb et al., reported that there was a statistically significant difference in unadjusted mortality rates between the BMS (5%) and DES (3.8%) [26]. Also, in the study by Dieguez et al. [27], the rate of all-cause mortality in patients treated with DES (6.5%) was significantly lower than that of patients treated with BMS (12.2%) (*P* = 0.049).

The study conducted by Melberg et al., showed that after a median of 10 years' follow-up, a quarter of the patients were dead, and more than half of the patients died from non-cardiac causes. Also, causes of death will change from MACE (MACCE) and be more dominated by cancer, especially after 5 years [28]. However, in the present study, cancer was the cause of death in only four patients.

One of the limitations of the present study is that it may be difficult to confirm the cause of death for people who died out of hospital. Since fewer diagnostic tests in terminally ill or elderly patients may be performed, the causes listed in the death certificates may be inconclusive. Also, the analysis of this type of data with composite endpoints from a competing risk perspective can be considered.

## Conclusion

The current study showed that the use of machine-learning prediction models such as RSF may improve long-term prediction in patients undergoing coronary stenting. Although the prediction performance of RSF based on the prediction error criteria was better than the Cox model, the most important variables identified in the two methods were similar. Our findings imply that the presentation of coronary artery disease (acute or chronic) and hyperlipidemia may also be considered as important prognostic variables in addition to diabetes, smoking, age, and stent length. The risk of complications may be modified by controlling these prognostic factors.

## Data Availability

The datasets during and/or analyzed during the current study are available from the corresponding author on reasonable request.

## Abbreviations

CABG: Coronary artery bypass graft surgery
CAD: Coronary artery disease
DES: Drug-eluting stent
BMS: Bare-metal stents
MACCE: Major adverse cardiac and cerebrovascular events
MI: Myocardial infarction
PCI: Percutaneous coronary intervention
TVR: Target vessel revascularization
RSF: Random survival forest

## References

[1] Kim AS, Johnston SC (2011). Global variation in the relative burden of stroke and ischemic heart disease. Circulation.

[2] Athappan G, Ponniah T (2009). Clinical outcomes of dialysis patients after implantation of DES: meta-analysis and systematic review of literature. Miner Cardioangiol.

[3] Jukema JW, Verschuren JJ, Ahmed TA, Quax PH (2011). Restenosis after PCI. Part 1: pathophysiology and risk factors. Nat Rev Cardiol.

[4] Kim MS, Dean LS (2011). In-stent restenosis. Cardiovasc Ther.

[5] Ashrith G, Elayda MA, Wilson JM (2010). Revascularization options in patients with chronic kidney disease. Tex Heart Inst J.

[6] Kleinbaum DG (1998). Survival analysis, a self-learning text. Biometrical J.

[7] Cox D (1972). Regression models and life-tables. J R Stat Soc.

[8] Breiman L (2001). Random forests. Mach Learn.

[9] Meng J, Li P, Zhang Q, Yang Z, Fu S (2014). A four-long noncoding RNA signature in predicting breast cancer survival. J Exp Clin Cancer Res.

[10] Noori S, Nourijelyani K, Mohammad K, Niknam M, Mahmoudi M, Andonian L (2012). Random forests analysis: a modern statistical method for screening in high-dimensional studies and its application in a population-based genetic association study. J North Khorasan Univ Med Sci.

[11] Kawaguchi A, Yajima N, Tsuchiya N, Homma J, Sano M, Natsumeda M (2013). Gene expression signature-based prognostic risk score in patients with glioblastoma. Cancer Sci.

[12] Miao F, Cai YP, Zhang YT, Li CY. Is random survival forest an alternative to Cox proportional model on predicting cardiovascular disease? In 6th European conference of the international federation for medical and biological engineering; 2015. Springer.

[13] Trikalinos TA, Alsheikh-Ali AA, Tatsioni A, Nallamothu BK, Kent DM (2009). Percutaneous coronary interventions for non-acute coronary artery disease: a quantitative 20-year synopsis and a network meta-analysis. Lancet.

[14] Shwaran H, Kogalur UB (2007). Random survival forests for R. R News.

[15] Brier GW (1950). Verification of forecasts expressed in terms of probability. Month Weather Rev.

[16] Aghajani H, Nezami P, Shafiee A, Jalali A, Nezami A, Nozari Y, Pourhosseini H (2018). Predictors of long-term major adverse cardiac events following percutaneous coronary intervention in the elderly. Arch Iran Med.

[17] Zhou Y, Zhu R, Chen X, Xu X, Wang Q, Jiang L (2019). Machine learning-based cardiovascular event prediction for percutaneous coronary. JACC Cardiovasc Interv.

[18] Meliga E, Garcia-Garcia HM, Valgimigli M, Biondi-Zoccai G, O.Maree A (2008). Longest available clinical outcomes after drug-eluting stent implantation for unprotected left main coronary artery disease: the DELFT (drug eluting stent for LeFT main) registry. JACC Cardiovasc Interv.

[19] Ebrahimzadeh F, Salehi Veisi M, Hajizadeh E, Namdari M (2018). Prediction of coronary artery restenosis in patients undergoing angioplasty. J Babol Univ Med Sci.

[20] Ebrahimzadeh F, Hajizadeh E, Baghestani A, Nasseryan J (2017). Timing the incidence of restenosis and some effective factors in patients undergoing angioplasty using extended cox regression model. J Mazandaran Univ Med Sci.

[21] Farshidi H, Abdi A, Madani A, Moshiri Sh, Ghasemi A, Hakimian R (2018). Major adverse cardiovascular event (MACE) after percutaneous coronary intervention in one-year follow-up study. Electron Phys.

[22] Tsai IT, Wang CP, Lu YC, Hung YC, Wu CC, Lu LF (2017). The burden of major adverse cardiac events in patients with coronary artery disease. BMC Cardiovasc Disord.

[23] Horst B, Rihal CS, Holmes DR, Bresnahan JF, Prasad A, Gau G (2016). Drug-eluting or bare-metal stents for coronary artery disease. N Engl J Med.

[24] De Felice F, Fiorilli R, Parma A, Nazzaro M, Musto C, Sbraga F (2009). 3-year clinical outcome of patients with chronic total occlusion treated with drug-eluting stents. JACC Cardiovasc Interv.

[25] Cai A, Dillon Ch, Hillegass WB, Beasley M, Brott BC, Bittner VA (2019). Risk of major adverse cardiovascular events and major hemorrhage among white and black patients undergoing percutaneous coronary intervention. J Am Heart Assoc.

[26] Duggal B, Subramanian J, Duggal M, Singh P, Rajivlochan M, Saunik S (2018). Survival outcomes post percutaneous coronary intervention: why the hype about stent type? Lessons from a healthcare system in India. PLoS ONE.

[27] Diéguez AR, Cid Álvarez AB, Nouche RT, Ávila Carrillo A, Álvarez Álvarez B, Gómez Peña F (2019). Drug-eluting versus bare-metal stents in primary PCI. Analysis of an 8-year registry. REC Interv Cardiol.

[28] Melberg T, Kjell Nygard O, Kier-Jan Kuiper K, Nordrehaug JE (2010). Competing risk analysis of events 10 years after revascularization. Scand Cardiovasc J.

